# SDMA attenuates renal tubulointerstitial fibrosis through inhibition of STAT4

**DOI:** 10.1186/s12967-023-04181-9

**Published:** 2023-05-16

**Authors:** Yanzhe Wang, Ming Wu, Dongping Chen, Bo Tan, Pinglan Lin, Di Huang, Chaoyang Ye

**Affiliations:** 1 Department of Nephrology, Shuguang Hospital Affiliated to Shanghai University of Traditional Chinese Medicine, No.528 Zhangheng Road, Pudong District, Shanghai, 201203 People’s Republic of China; 2 TCM Institute of Kidney Disease of Shanghai University of Traditional Chinese Medicine, Shanghai, China; 3 Key Laboratory of Liver and Kidney Diseases, Ministry of Education, Shanghai Key Laboratory of Traditional Chinese Clinical Medicine, Shanghai, China; 4 Clinical Pharmacokinetic Laboratory, Shuguang Hospital Affiliated to Shanghai University of Traditional Chinese Medicine, Shanghai, China

**Keywords:** SDMA, Renal fibrosis, STAT4

## Abstract

**Background:**

Renal tubulointerstitial fibrosis is the hallmark of various chronic kidney diseases. Symmetric dimethylarginine (SDMA) is an independent cardiovascular risk factor in patients with chronic kidney diseases, which is mostly excreted through renal tubules. However, the effect of SDMA on kidneys in a pathological condition is currently unknown. In this study, we investigated the role of SDMA in renal tubulointerstitial fibrosis and explored its underlying mechanisms.

**Methods:**

Mouse unilateral ureteral obstruction (UUO) and unilateral ischemia–reperfusion injury (UIRI) models were established to study renal tubulointerstitial fibrosis. SDMA was injected into kidneys through ureter retrogradely. TGF-β stimulated human renal epithelial (HK2) cells were used as an in vitro model and treated with SDMA. Signal transducer and activator of transcription-4 (STAT4) was inhibited by berbamine dihydrochloride or siRNA or overexpressed by plasmids in vitro. Masson staining and Western blotting were performed to evaluate renal fibrosis. Quantitative PCR was performed to validate findings derived from RNA sequencing analysis.

**Results:**

We observed that SDMA (from 0.01 to 10 µM) dose-dependently inhibited the expression of pro-fibrotic markers in TGF-β stimulated HK2 cells. Intrarenal administration of SDMA (2.5 µmol/kg or 25 µmol/kg) dose-dependently attenuated renal fibrosis in UUO kidneys. A significant increase in SDMA concentration (from 19.5 to 117.7 nmol/g, p < 0.001) in mouse kidneys was observed after renal injection which was assessed by LC–MS/MS. We further showed that intrarenal administration of SDMA attenuated renal fibrosis in UIRI induced mouse fibrotic kidneys. Through RNA sequencing analysis, we found that the expression of STAT4 was reduced by SDMA in UUO kidneys, which was further confirmed by quantitative PCR and Western blotting analysis in mouse fibrotic kidneys and renal cells. Inhibition of STAT4 by berbamine dihydrochloride (0.3 mg/ml or 3.3 mg/ml) or siRNA reduced the expression of pro-fibrotic markers in TGF-β stimulated HK2 cells. Furthermore, blockage of STAT4 attenuated the anti-fibrotic effect of SDMA in TGF-β stimulated HK2 cells. Conversely, overexpression of STAT4 reversed the anti-fibrotic effect of SDMA in TGF-β stimulated HK2 cells.

**Conclusion:**

Taken together, our study indicates that renal SDMA ameliorates renal tubulointerstitial fibrosis through inhibition of STAT4.

**Supplementary Information:**

The online version contains supplementary material available at 10.1186/s12967-023-04181-9.

## Highlights

- SDMA attenuates renal tubulointerstitial fibrosis in fibrotic kidneys
- SDMA has a direct anti-fibrotic effect on renal epithelial cells
- Inhibition of STAT4 prevents EMT in renal epithelial cells
- SDMA inhibits EMT and renal tubulointerstitial fibrosis through targeting STAT4

**Supplementary Information:**

The online version contains supplementary material available at 10.1186/s12967-023-04181-9.

## Background

Chronic kidney diseases (CKD) affect more than 10% of world population causing a heavy social and economic burden [1]. Renal tubulointerstitial fibrosis is the most important pathological feature of various CKD and is the best predictor for renal function outcomes [2, 3]. Hallmarks of renal fibrosis include overexpression of extracellular matrix (ECM) fibrils (collagen-I, fibronectin, etc.), up-regulation of epithelial to mesenchymal transition (EMT) markers (α-SMA, N-cadherin, Snail, etc.), and activation of TGFβ/Smad3 signaling pathway [4–6].

Besides the classic TGFβ/Smad3 signaling pathway, multiple signaling pathways are dysregulated in fibrotic kidneys. Impaired peroxisome proliferative activated receptor alpha (PPARA), hepatocyte nuclear factor 4 alpha (HNF4A) and activation of nuclear factor kappa-B (NFκB, such as c-Rel), Sex determining region Y-Box 6 (SOX6), early growth response protein 2 (EGR2), c-Myb signaling pathways are associated with progression of renal fibrosis [7–13]. Krüppel-like factors (KLFs) belong to a family of zinc-finger transcription factors, which is a research hotspot in the field of kidney disease and several KLF family members have been identified as important players in renal tubulointerstitial fibrosis [14]. STAT4 is a well-known regulator involved in autoimmunity and inflammation [15, 16]. Since renal fibrosis is characterized by activation of inflammatory responses, STAT4 signaling pathway may also be involved in renal fibrosis [17].

Asymmetrical dimethylarginine (ADMA) and symmetric dimethylarginine (SDMA) are both byproducts of protein methylation, and both of them are increased in the circulation of patients with end stage renal disease (ESRD) [18–20]. Not like ADMA, SDMA is not a death predictor in ESRD [20]. However, SDMA is still regarded as a risk factor for cardiovascular events [18]. This is probably due to that SDMA is a well-known inducer of endothelial damages and thus endothelial-related disease is the focus of SDMA study so far [21, 22]. The majority of SDMA is excreted through the kidney [19]. Interestingly, chronic infusion of SDMA has no detrimental effect on normal kidneys [23]. However, the effect of SDMA on kidney in a pathological condition has not been studied. The proximal tubule is the most vulnerable target of kidney injuries [24]. The role of SDMA in tubular associated kidney disease is currently unknown.

In this study, we aimed to study the role of SDMA in chronic kidney disease with a focus on renal tubulointerstitial fibrosis and explored its underlying mechanisms.

## Methods

### Animal studies

Wild type male C57BL/6 mice with body weight between 20 and 25 g were purchased from Shanghai SLAC Laboratory Animal Co., Ltd and housed in a SPF grade animal facility in the Shanghai University of Traditional Chinese Medicine under the local regulations.

UUO surgery was performed through twice ligation of the left ureter with 4–0 nylon sutures as described in a previous study [25]. Mice were randomly divided into five groups: (I) UUO + normal saline (NS, n = 10), (II) UUO + 2.5 µmol/kg symmetric dimethylarginine (SDMA, n = 10), (III)UUO + 25 µmol/kg SDMA (n = 10) group. In another experiment, sham operated mice were divided into three groups: (I) sham + no renal injection (n = 10), (II) sham + normal saline (n = 10), (III)sham + 25 µmol/kg SDMA (n = 10) group. Mice were sacrificed at day7 to collect renal tissue.

To measure the content of renal SDMA after intrarenal injection, UUO mice were injected with normal saline (n = 5) or 25 µmol/kg SDMA (n = 5). Mice were sacrificed at 1 h after the injection.

For the unilateral ischemia–reperfusion injury (UIRI) model, left renal pedicles were clamped for 35 min by using microaneurysm clamps in male mice. Mice were randomly divided into three groups: (1) UIRI + normal saline (n = 10), (2) UIRI + 25 µmol/kg SDMA (n = 10). Mice were sacrificed at day 11 for tissue collection.

### Intrarenal SDMA administration

SDMA (T7344) was purchased from TargetMol Chemicals Inc. (Boston, MA, USA) and dissolved in normal saline as a 100 mM stock, which was further diluted with normal saline to working solution at different concentrations. 0.04% typan blue dye (A601140, Sangon, Shanghai, China) was added into the working solution to monitor the injection process. 50 μL of normal saline, 1 mM or 10 mM SDMA (2.5 µmol/kg or 25 µmol/kg) was injected retrogradely once into the left kidney via the ureter. Unilateral ureteral obstruction was performed after the injection. In UIRI mouse model, 50 μL of normal saline or 10 mM SDMA (25 µmol/kg) was injected into the left kidney via the ureter, which was clamped by using a microaneurysm clamps just before the ischemia surgery. After 35-min ischemia, ureter and renal artery clamps were released. The dosage of SDMA that we used in this study was calculated by considering a previous publication where SDMA (250 µmol/kg/day) was systemically infused into mice by using osmotic minipumps for a long-term [23]. In sham operated mice, the left ureter was clamped for 30 min after normal saline or SDMA injection.

### Quantitation of renal SDMA and asymmetrical dimethylarginine (ADMA)

Renal concentrations of SDMA and ADMA were measured by using a validated LC–MS/MS method with minor modification [26]. In brief, kidney tissue samples were added 10 volumes of ice-cold normal saline (10 ml/g wet weight of tissue) and homogenized immediately. The kidney homogenates (50 μl) were mixed with acetonitrile-methanol mixture (1: 1, v/v) to which stable isotope-labelled internal standard (2,3,3,4,4,5,5-d_7_-ADMA) had been added. Then, those compounds were converted into their butyl ester derivatives and measured by a Waters LC–MS/MS system, which contained an ACQUITY UPLC and an Xevo TQ-S tandem quadrupole mass spectrometry (Waters, Milford, MA, USA). Compounds were separated on an Acquity UPLC BEH C_18_ column (2.1 × 100 mm, 1.7 µm, Waters). The coefficients of variation for both ADMA and SDMA were below 15%.

### Cell culture

HK2 renal proximal tubular epithelial cells were purchased from the Cell Bank of Shanghai Institute of Biological Sciences (Chinese Academy of Science). Cells were seeded in 6-well plate to 40–50% confluence, which were starved overnight with DMEM/F12 medium containing 0.5% fetal bovine serum (FBS). On the next day, cells were changed with fresh 0.5% medium and exposed to 2.5 ng/ml TGF-β (Peprotech, Rocky Hill, NJ, USA) for 48 h in the presence of various concentration of SDMA or STAT4 inhibitor berbamine dihydrochloride (T2920, TargetMol).

Nonsense control (NC) or human STAT4 siRNA were transfected by Lipofectamine 2000 (11668–027; Invitrogen) in HK2 cells by using DMEM/F12 medium containing 10% fetal bovine serum according to the manufacturer’s instruction. After 48 h transfection, protein was extracted from cells. In another experiment, fresh medium containing 0.5% fetal bovine serum was changed in the second day after transfection, and cells were exposed to 2.5 ng/ml TGF-β for 48 h with or without SDMA. The NC siRNA sequences were as follows: forward, 5′-UUCUCCGAACGUGUCACGUTT -3′; and reverse, 5′-ACG UGACACGUUCGGAGAATT -3′. The human STAT4 siRNA sequences were as follows: forward, 5′-GCUGUUGCUAAAGGAUAAATT -3′; and reverse, 5′-UUUAUCCUUUAGCAACAGCTT -3′.

Empty vector (pENTER) and human STAT4 plasmid were purchased from WZ biosciences (Jinan, China) and transfected by Lipofectamine 2000 (11668–027; Invitrogen) in HK2 cells by using DMEM/F12 medium containing 10% fetal bovine serum according to the manufacturer’s instruction. On the next day cells were stimulated with TGF-β and treated with 10 µM SDMA. After another 24 h, protein was extracted from cells.

### Masson’s trichrome

Mouse kidneys were sliced, fixed, and embedded in paraffin, and cut into 4 μm-thick sections. The paraffin-embedded kidney section was stained with hematoxylin, and then with ponceau red liquid dye acid complex, which was followed by incubation with phosphomolybdic acid solution. Finally, the tissue was stained with aniline blue liquid and acetic acid. Images were captured by using a microscope (Nikon 80i, Tokyo, Japan).

### RNA-seq analysis

Total RNA was isolated from mouse kidneys by using TRIzol^®^ Reagent (Invitrogen) and genomic DNA was removed by using DNase I (TaKara). RNA quality was determined by 2100 Bioanalyser (Agilent) and quantified by ND-2000 (NanoDrop Technologies). RNA sample with high-quality (OD260/280 = 1.8 ~ 2.2, OD260/230 ≥ 2.0, RIN ≥ 6.5, 28S:18S ≥ 1.0, > 1 µg) was used to construct sequencing library.

RNA-seq transcriptome library was prepared from 1 μg of total RNA by Majorbio biotech company (Shanghai, China) by using TruSeqTM RNA sample preparation Kit (Illumina, San Diego, CA). Paired-end RNA-seq sequencing library was sequenced with the Illumina HiSeq xten/NovaSeq 6000 sequencer (2 × 150 bp read length). Low quality reads were removed from the raw RNA-seq reads by using by SeqPrep (https://github.com/jstjohn/SeqPrep) and Sickle (https://github.com/najoshi/sickle) with default parameters. Clean reads were aligned to reference genome with orientation mode by using HISAT2 (http://ccb.jhu.edu/software/hisat2/index.shtml) software. The mapped reads of each sample were assembled by StringTie (https://ccb.jhu.edu/software/stringtie/index.shtml?t=example) in a reference-based approach.

Differentially expressed genes (DEGs) were calculated by DESeq2/DEGseq/EdgeR with Q ≤ 0.05. DEGs with |log2FC|> 1 and Q ≤ 0.05(DESeq2 or EdgeR) /Q ≤ 0.001(DEGseq) were significantly different expressed genes. Functional-enrichment analysis including gene ontology (GO) were performed to identify which DEGs were significantly enriched in GO terms with Bonferroni-corrected P ≤ 0.05, when compared with the whole-transcriptome background. GO functional enrichment analysis were carried out by Goatools (https://github.com/tanghaibao/Goatools). All raw data have been deposited under the Gene Expression Omnibus accession number PRJNA783810.

### Quantitative PCR (qPCR)

Total RNA was extracted by using Trizol (R401-01, Vazyme, Nanjing, China) from kidney samples according to the manufacture’s instruction, which was reverse transcribed to cDNA by Takara PrimeScript RT reagent kit (RR0036A, Kyoto, Japan). The primer sequences for qPCR were listed in Additional file 2: Table S1. Hieff Unicon^®^ qPCR TaqMan Probe Master Mix (11205E, Yeasen Biotechnology, Shanghai, China) was used. qPCR was performed by using a StepOne Plus Sequence Detection System (Applied Biosystems). All gene expression levels were normalized to Gapdh, and results are expressed as fold change in mRNA expression.

### Western blotting analysis

Cell or kidney protein was extracted by using lysis buffer bought from Beyotime Biotech (Nantong, China). BCA Protein Assay Kit (P0012S, Beyotime Biotech, Nantong, China) was used to determine the protein concentration. Protein samples were dissolved in 5 × SDS-PAGE loading buffer (P0015L, Beyotime Biotech, Nantong, China), which were further subjected to SDS-PAGE gel electrophoresis. After electrophoresis, proteins were electro-transferred to a polyvinylidene difluoride membrane (Merck Millipore, Darmstadt, Germany). Unspecific binding on the PVDF membrane was blocked by incubation with the blocking buffer (5% non-fat milk, 20 mM Tris–HCl, 150mMNaCl, PH = 8.0, 0.01%Tween 20) for 1 h at room temperature, which was followed by incubation with anti-fibronectin (1:1000, ab23750, Abcam), anti-pSmad3 (1:1000, ET1609-41, HUABIO), anti-Collagen I (1:500, sc-293182, Santa Cruz), anti-α-SMA (1:1000, ET1607-53, HUABIO), anti-N-cadherin (1:1000, sc-59887, Santa Cruz), anti-Snail (1:1000, A11794, Abclonal), anti-STAT4 (1:1000, ET1701-42, HUABIO), anti-GAPDH (1:5000, 60004-1-lg, Proteintech) antibodies overnight at 4 ℃. Binding of the primary antibody was detected by an enhanced chemiluminescence method (SuperSignal^™^ West Femto, 34,094, Thermo Fisher Scientific) by using horseradish peroxidase-conjugated secondary antibodies (goat anti-rabbit IgG, 1:1000, A0208, Beyotime or goat anti-mouse IgG, 1:1000, A0216, Beyotime).

### Statistical analysis

Results were presented as mean ± SD. Differences among multiple groups were analyzed by one-way analysis of variance (ANOVA) and comparison between two groups was performed by unpaired student t-test by using GraphPad Prism version 8.0.0 for Windows (GraphPad Software, San Diego, California USA). A P value of lower than 0.05 was considered statistically significant.

## Results

### SDMA inhibits the expression of pro-fibrotic markers in renal cells

**Fig. 1:**
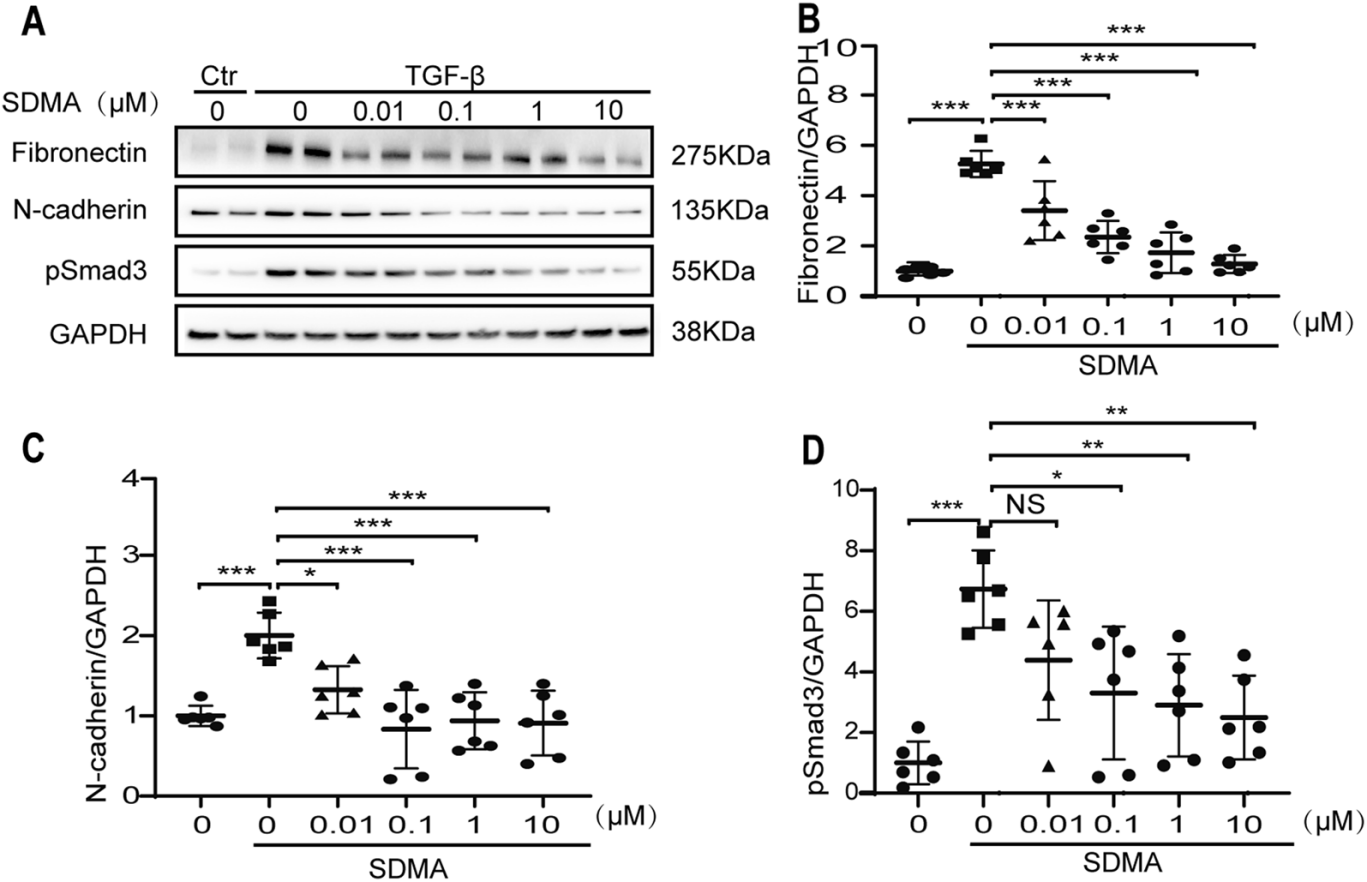
Symmetric dimethylarginine (SDMA) inhibits the expression of pro-fibrotic markers in renal cells. HK2 human renal epithelial cells were starved overnight and followed by stimulation with TGF-β and treatment with different concentration (0.01–10 µM) of SDMA for 48 h. Western blotting (**A**) was performed to analyze the expression of fibronectin (**B**), N-cadherin (**C**) and phosphorylated Smad3 (pSmad3) (**D**). Data represents mean ± SD. One representative result of at least three independent experiments is shown. NS represents not significant. *p < 0.05. **p < 0.01. ***p < 0.001

The effect of SDMA on renal fibrosis was first studied by using TGF-β stimulated human renal epithelial (HK2) cells. As shown in Fig. 1, SDMA dose-dependently inhibited the expression of fibronectin, N-cadherin and phosphorylated Smad3 (pSmad3) in TGF-β stimulated HK2 cells from 0.01 µM or 0.1 µM to 10 µM, suggesting that SDMA is anti-fibrotic in renal epithelial cells.

### SDMA inhibits renal tubulointerstitial fibrosis in unilateral ureter obstruction (UUO) kidneys

**Fig. 2:**
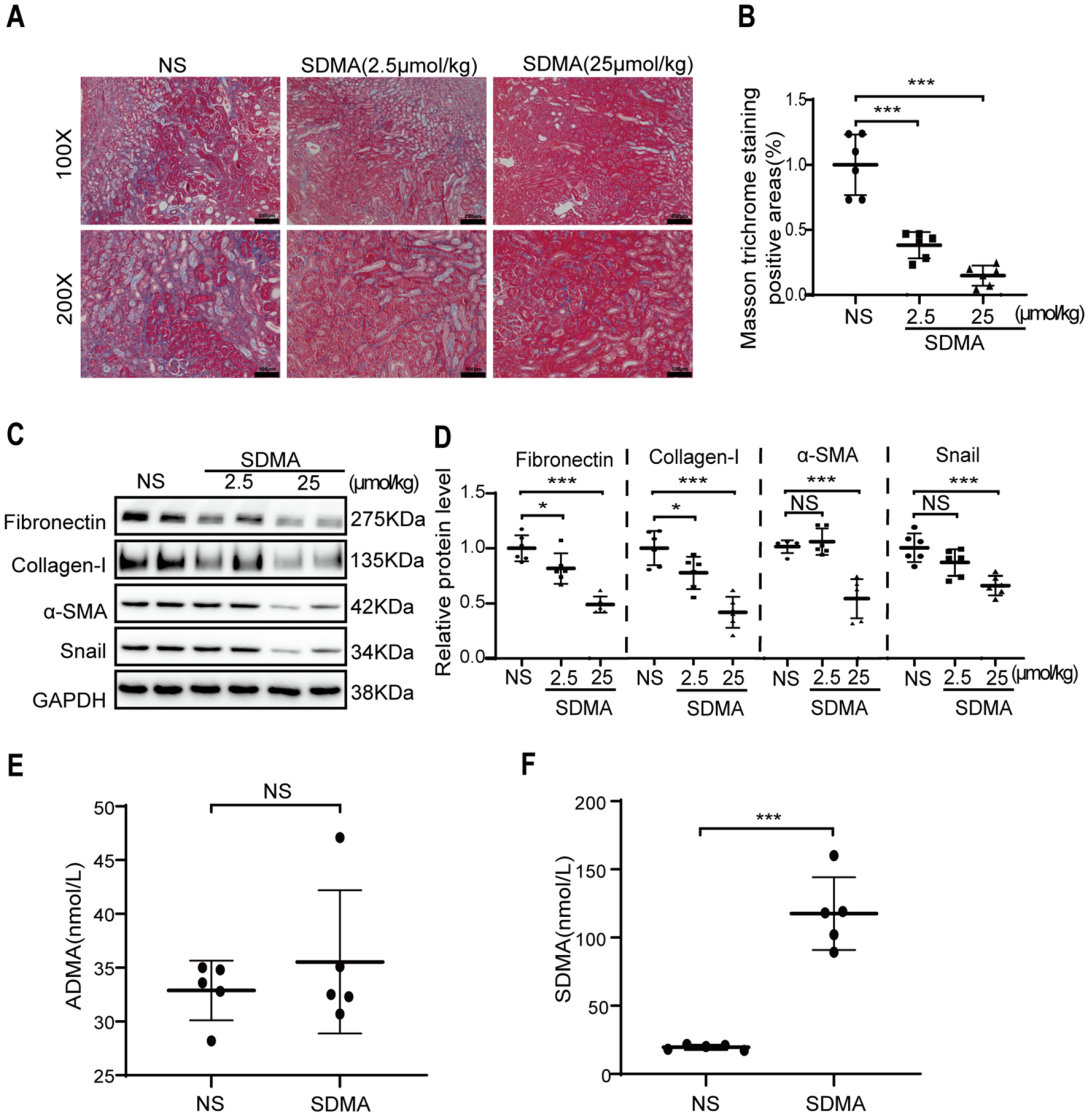
Renal injection of SDMA attenuates renal tubulointerstitial fibrosis in unilateral ureteral obstruction (UUO) kidneys. Male c57 mice received UUO operation and were sacrificed at day 7. Renal injection of 50 μL of normal saline (NS), 1 mM or 10 mM SDMA (2.5 µmol/kg or 25 µmol/kg) was performed during UUO operation. **A** Renal fibrosis was assessed by Masson’s trichrome staining and quantified **B**. Scale bar = 200 µm for upper figures and scale bar = 100 µm for lower figures. The expression of fibronectin, collagen-I, α-SMA and Snail were analyzed by Western blotting (**C**) and quantified (**D**). One representative of at least three independent experiments is shown. Renal injection of 50 μL of normal saline (NS) or SDMA (25 µmol/kg) was performed right before UUO operation. Renal tissue was collected at 1 h after the operation. The concentration of renal asymmetrical dimethylarginine (ADMA) (**E**) and SDMA (**F**) were measured by using a LC–MS/MS method. Data represent mean ± SD. NS represents not significant. *p < 0.05. ***p < 0.001

We further studied the role of SDMA in renal tubulointerstitial fibrosis by using the UUO mouse model. Massive interstitial extracellular matrix deposition was observed in normal saline (NS) treated UUO kidneys at 7 days after the operation as shown by Masson staining, and intrarenal delivery of (2.5 µmol/kg or 25 µmol/kg) SDMA dose-dependently reduced collagen deposition (Fig. 2A–B). Increased expression of ECM proteins (fibronectin and collagen-I) and EMT markers (α-SMA and Snail) were observed in NS treated UUO kidneys by Western blotting analysis, which were dose-dependently reduced by SDMA treatment (Fig. 2C–D).

To exclude the effect of administration of a ureteral fluid bolus on renal fibrosis and the toxic effect of SDMA on healthy kidneys, we also injected normal saline or SDMA into sham kidneys. Masson staining or Western blotting analysis of the expression of fibrotic markers (fibronectin, collagen-I and α-SMA) showed no detrimental effect of administration of NS or SDMA on sham kidneys (Additional file 1: Figure S1).

Retrograde ureteral injection is a well-established technique for kidney injection and used to transfect renal tubular cells with virus [27–29]. To confirm that SDMA was indeed injected into kidneys, the concentration of renal asymmetrical dimethylarginine (ADMA) and SDMA at 1 h after intrarenal injection SDMA (25 µmol/kg) was measured by LC–MS/MS. The concentration of renal ADMA was unchanged (32.9 nmol/g vs 35.5 nmol/g) after renal injection of SDMA as shown in Fig. 2E. As shown in Fig. 2F, renal SDMA was significantly increased from 19.5 nmol/g to 117.7 nmol/g after intrarenal injection with 25 µmol/kg SDMA.

### SDMA inhibits renal tubulointerstitial fibrosis in unilateral ischemia–reperfusion injury (UIRI) kidneys

**Fig. 3:**
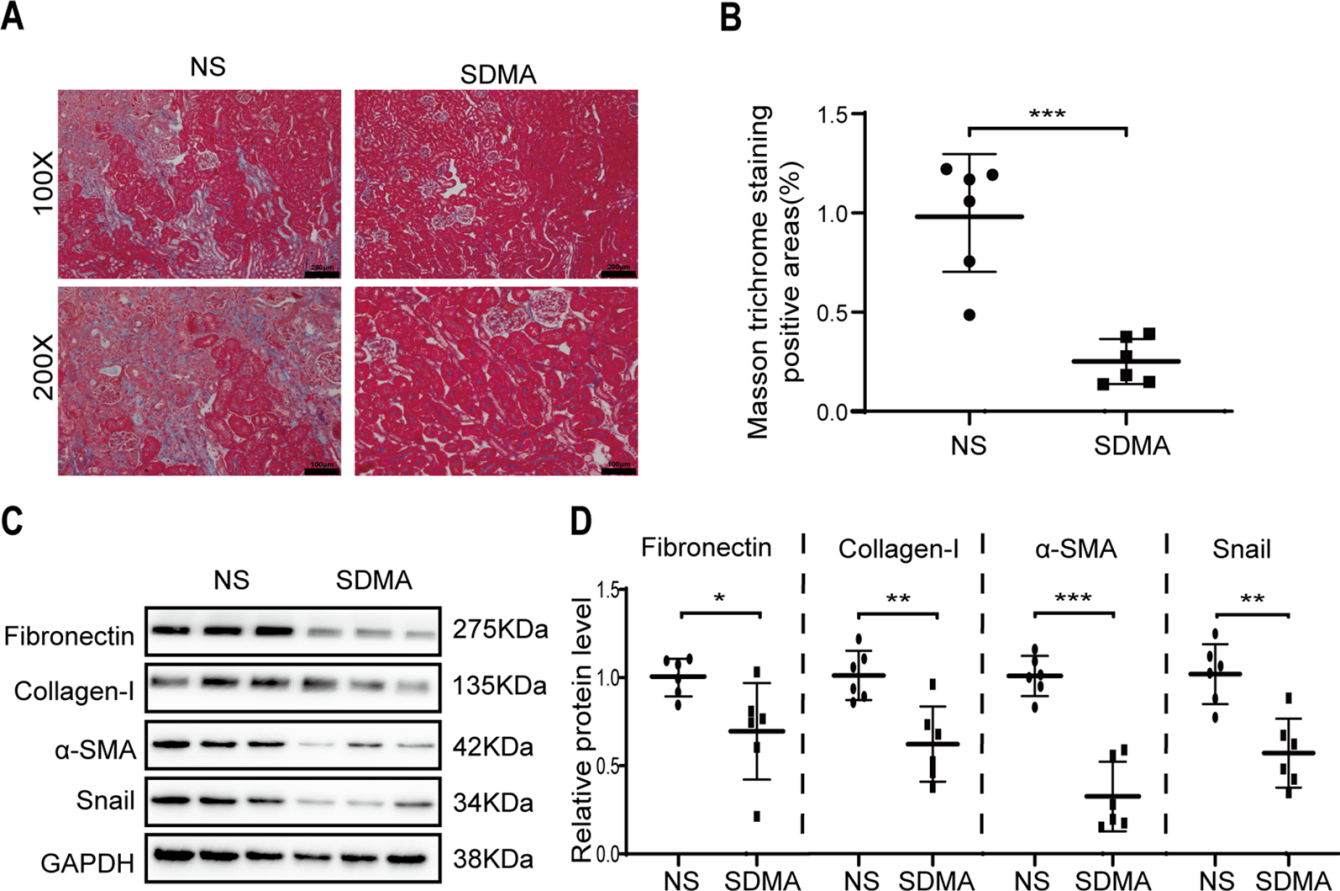
Administration of SDMA attenuates renal tubulointerstitial fibrosis in unilateral ischemia–reperfusion injury (UIRI) kidneys. Male c57 mice received UIRI operation for 35 min and were sacrificed at day 11. Renal injection of 50 μL of NS or 10 mM SDMA (25 µmol/kg) was performed during UIRI operation. Renal fibrosis was assessed by Masson’s trichrome staining (**A**) and quantified (**B**). Scale bar = 200 µm for upper figures and scale bar = 100 µm for lower figures. The expression of fibronectin, collagen-I, α-SMA and Snail were analyzed by Western blotting (**C**) and quantified (**D**). One representative of at least three independent experiments is shown. Data represent mean ± SD. ND represents not determined. *p < 0.05. **p < 0.01. ***p < 0.001

To further confirmed the role of SDMA in renal tubulointerstitial fibrosis, we used another model of renal tubulointerstitial fibrosis. Mild interstitial ECM deposition was observed in UIRI kidneys at 11 days after the UIRI operation as shown by Masson staining, and intrarenal administration of SDMA reduced ECM deposition (Fig. 3A–B). UIRI operation enhanced the expression of ECM proteins (fibronectin and collagen-I) and EMT markers (ɑ-SMA and Snail) in mouse kidneys as shown by Western blotting analysis, which were reduced by 25 µmol/kg SDMA treatment (Fig. 3C–D).

### SDMA inhibits STAT4 expression in fibrotic kidneys

**Fig. 4:**
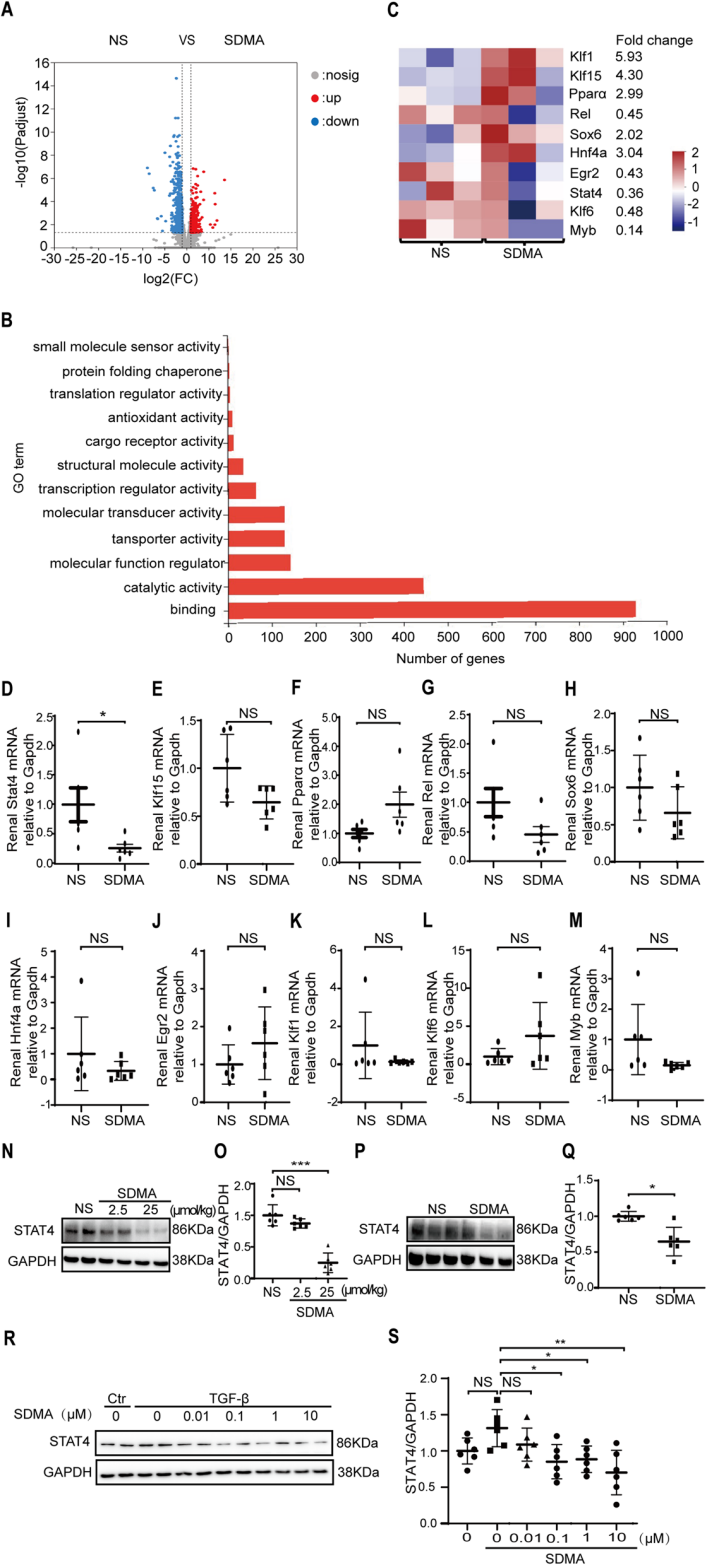
SDMA inhibits STAT4 expression in fibrotic kidneys. Volcano plots showing differentially expressed genes between NS and SDMA injected mice upon UUO operation, which was identified by RNA sequencing (RNA-seq) (**A**). Gene ontology (GO) function enrichment analysis of SDMA downstream genes (**B**). Heatmap of expression values of genes enriched in the category of transcription regulator activity (**C**). Gene expression values are displayed by applying progressively brighter shades of red (up-regulated) or blue (down-regulated). Quantitative PCR analysis of mRNA levels of representative SDMA downstream genes in kidneys between NS and SDMA treated mice upon UUO operation (**D**–**M**). The expression of STAT4 were analyzed by Western blotting and quantified in UUO (**N**–**O**) and UIRI (**P**–**Q**) kidneys treated with NS or SDMA. The expression of STAT4 were analyzed by Western blotting and quantified in TGF-β stimulated HK2 renal epithelial cells in the presence of NS or various concentration of SDMA (**R**–**S**). These results are from three independent experiments. Data represent mean ± SD. NS represents not significant. *p < 0.05. **p < 0.01. ***p < 0.001

To examine the effect of SDMA on transcriptional profiles of fibrotic kidneys, we performed RNA sequencing (RNA-seq) in NS and SDMA injected UUO kidneys. Total 1281 differential expressed genes were identified by RNA-seq. Transcriptional profiles in NS and SDMA injected UUO kidneys differed substantially, with 559 genes downregulated and 722 genes upregulated over twofold (Log2FC ≥ 1) in SDMA treated UUO kidneys as compared with NS treated UUO kidneys (Fig. 4A).

The gene ontology (GO) enrichment analysis showed that the most significantly enriched molecular functions for the differentially expressed mRNAs in UUO kidneys were those associated with binding, catalytic activity, molecular function regulator, transporter activity, molecular transducer activity, transcriptional regulator activity, structural molecule activity, cargo receptor activity, antioxidant activity, and translation regulator activity (Fig. 4B). We focused on 63 differential expressed genes enriched in the category of transcription regulator activity. Next, we validated the expression of several transcriptional factors enriched in this category (*Klf1*; *Klf15*; *Ppara*, *Rel*, *Sox6*, *Hnf4a, Egr2, Stat4, Klf6, Myb*) by performing quantitative PCR (qPCR) assays (Fig. 4C–M). Only the differential expression of *Stat4* gene in NS and SDMA treated UUO kidneys was confirmed by qPCR with significant difference (Fig. 4D). Western blotting analysis was performed to assess the expression of *Stat4* in the mouse UUO model. Down-regulation of STAT4 in UUO kidneys by SDMA treatment was observed (Fig. 4N–O), which was further confirmed in UIRI kidneys (Fig. 4P–Q). We further showed that SDMA dose-dependently inhibited the expression of STAT4 in TGF-β stimulated HK2 cells (Fig. 4R–S).

### STAT4 mediates the anti-fibrotic effect of SDMA

**Fig. 5:**
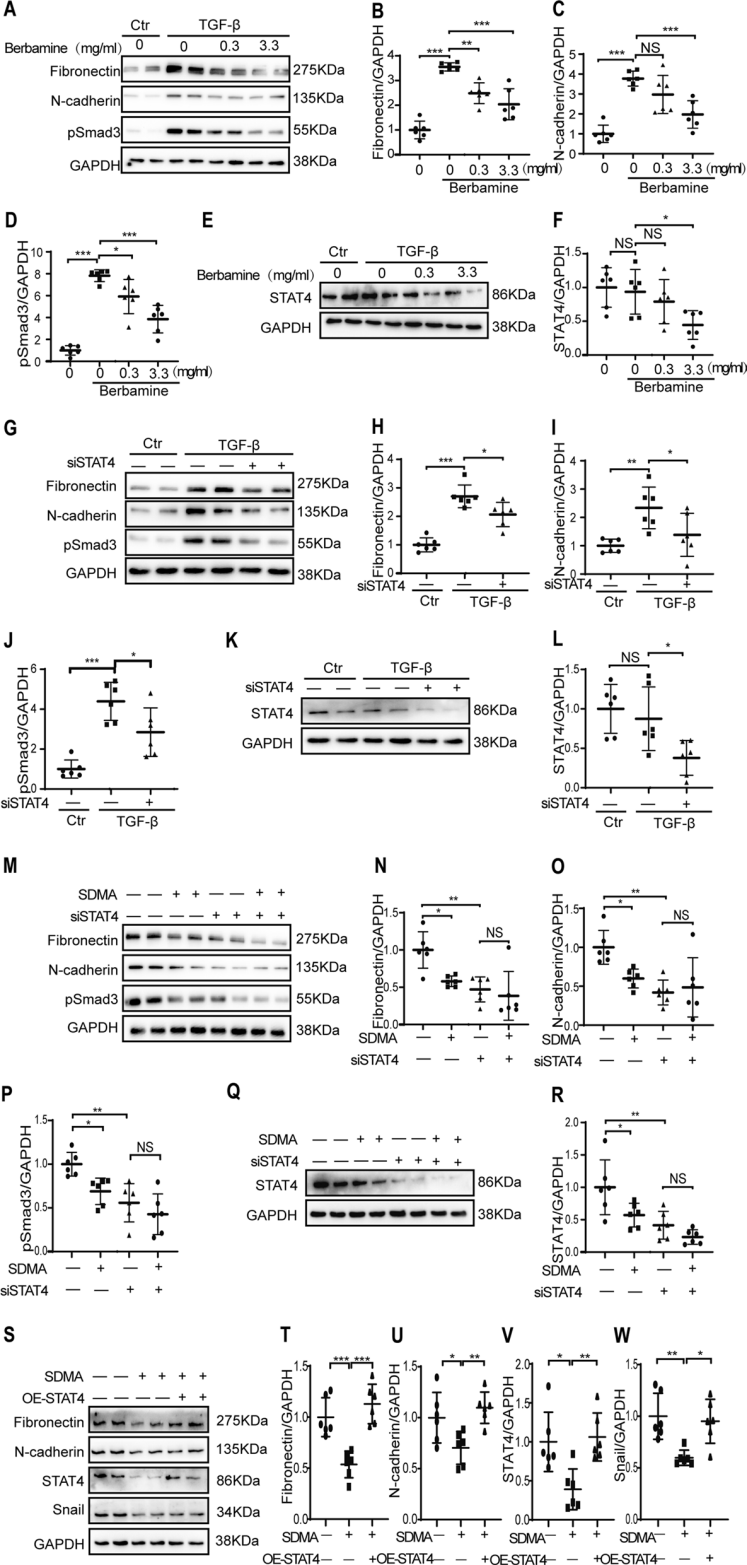
SDMA reduces the expression of pro-fibrotic markers in renal cells through STAT4. HK2 human renal epithelial cells were starved overnight and followed by stimulation with TGF-β and treatment with different concentration (0.3–3.3 mg/ml) of STAT4 inhibitor berbamine dihydrochloride for 48 h. The expression of fibronectin, N-cadherin, pSmad3, and STAT4 were analyzed by Western blotting (**A**, **E**) and then quantified (**B**–**D**, **F**). HK2 human renal epithelial cells were transfected with nonsense control or STAT4 siRNA (siSTAT4), followed by overnight starvation at 6 h after transfection. On the next day cells were stimulated with TGF-β for another 48 h. The expression of fibronectin, N-cadherin, pSmad3, and STAT4 were analyzed by Western blotting (**G**, **K**) and then quantified (**H**–**J**, **L**). HK2 human renal epithelial cells were transfected with nonsense control or STAT4 siRNA, followed by overnight starvation at 6 h after transfection. On the next day cells were stimulated with TGF-β and treated with 10 µM SDMA for another 48 h. The expression of fibronectin, N-cadherin, pSmad3, and STAT4 were analyzed by Western blotting (**M**, **Q**) and then quantified (**N**–**P**, **R**). HK2 human renal epithelial cells were transfected with empty vector or STAT4 plasmid (overexpression, OE). On the next day, cells were stimulated with TGF-β and treated with 10 µM SDMA for another 24 h. The expression of fibronectin, N-cadherin, Snail, and STAT4 were analyzed by Western blotting (S) and then quantified (**T**–**W**). Data represents mean ± SD. One representative result of at least three independent experiments is shown. NS represents not significant. *p < 0.05. **p < 0.01. ***p < 0.001

Next, we investigated the role of STAT4 in renal tubulointerstitial fibrosis. As shown in Figs. 4C–5A, the STAT4 inhibitor berbamine dihydrochloride inhibited the expression of fibronectin, N-cadherin, pSmad3 and STAT4 in TGF-β stimulated HK2 cells at 0.3 mg/ml or 3.3 mg/ml, which was correlated with down-regulation of STAT4 (Fig. 5E–F). Moreover, transfection of STAT4 siRNA reduced the expression of the expression of fibronectin, N-cadherin, pSmad3 and STAT4 in TGF-β stimulated HK2 cells as compared with that in nonsense control siRNA transfected cells (Fig. 5G–L).

We further showed that deletion of STAT4 by siRNA abolished the inhibitory effect of SDMA on the expression of fibronectin, N-cadherin and pSmad3 in TGF-β stimulated HK2 cells (Fig. 5M–R). Furthermore, overexpression of STAT4 reversed the inhibitory effect of SDMA on the expression of fibronectin, N-cadherin and Snail in TGF-β stimulated HK2 cells as shown by Western blotting analysis (Fig. 5M–R).

All these data indciate that STAT4 signaling pathway mediates the anti-fibrotic effect of SDMA on renal epithelial cells.

## Discussion

SDMA is mostly eliminated through the kidney, and it has no effect on healthy kidneys [23]. Although circulating SDMA is regarded as biomarker of renal function decline in aging animals, the effect of SDMA on diseased kidneys is still not known [30]. In the current study, we used in vitro and in vivo models to study the effect of SDMA on renal tubulointerstitial fibrosis. We first showed that SDMA dose-dependently inhibited the expression of ECM protein, EMT marker and pSmad3 in TGF-β stimulated renal epithelial cells. Second, intrarenal injection of SDMA dose-dependently reduced ECM deposition and pro-fibrotic marker expression in UUO kidneys, which was further confirmed in UIRI kidneys. We concluded that SDMA is renal protective in CKD in terms of inhibition of renal tubulointerstitial fibrosis.

SDMA is a well-known inducer of endothelial dysfunction and cause endothelial-related diseases through a mechanism by which SDMA competes with arginine for cell transport and thus reducing the bioavailability of nitric oxide [18, 31]. SDMA is associated with diastolic dysfunction in patients with heart failure and is a risk factor of CVD in CKD patients [18, 32]. In glomerular endothelial cells, SDMA enhances oxidative stress and may lead to glomerular damages in kidney diseases [21]. It is known that SDMA is mostly excreted through renal tubules, however the effect of SDMA on renal epithelial cells was not studied. To our knowledge, this is the first study showing that SDMA is beneficial to renal epithelial cells in a fibrotic condition.

The mechanisms downstream of SDMA is still largely unknown. Through RNA-seq analysis, we found that SDMA inhibited the expression of STAT4 in UUO kidneys, which was further shown in another fibrotic mouse model. The direct inhibitory effect of SDMA on STAT4 expression in renal epithelial cells was further confirmed. Finally, we also found that STAT4 signaling pathway mediates the inhibitory effect of SDMA on EMT and ECM protein production in renal epithelial cells. Thus, STAT4 is a signaling pathway downstream of SDMA.

We can not exclude the direct effect of SDMA on other renal cell types. STAT4 is a risk factor for inflammatory diseases such as rheumatoid arthritis and lupus erythematosus, and is initially identified as an important immunoregulator, which regulates various cytokine or chemokine production by immune cells [15, 33, 34]. Thus, SDMA may inhibit renal tubulointerstitial fibrosis partly through inhibition of STAT4 mediated cytokine production or immune cell infiltration.

Partial EMT is a hallmark of tubulointerstitial fibrosis in chronic kidney disease [35]. The involvement of STAT4 in EMT has been reported recently. Several studies revealed that STAT4 is not only involved in tumor metastasis, but also promotes EMT and fibroblast proliferation [36, 37]. In this work, we showed that the expression of STAT4 is positively correlated with the expression of EMT markers and ECM proteins in fibrotic kidneys. Moreover, inhibition of STAT4 through pharmacological or genetic approaches in renal epithelial cells reduced the expression of EMT markers and ECM proteins. Thus, STAT4 signaling is involved in EMT and promotes renal tubulointerstitial fibrosis.

Clinical studies revealed that polymorphisms of STAT4 is associated with the severity of kidney disease such as IgA nephropathy, lupus nephritis and primary membranous glomerulonephritis, suggesting that the activation of STAT4 signaling pathway is involved in immune-mediated kidney diseases [38–40]. Renal tubulointerstitial fibrosis is characterized by infiltration of immune cells and activation of inflammatory responses [2]. Thus, it is interesting to study the association of STAT4 polymorphisms or activation of STAT4 with the degree of renal tubulointerstitial fibrosis in CKD patients.

## Conclusion

**Fig. 6:**
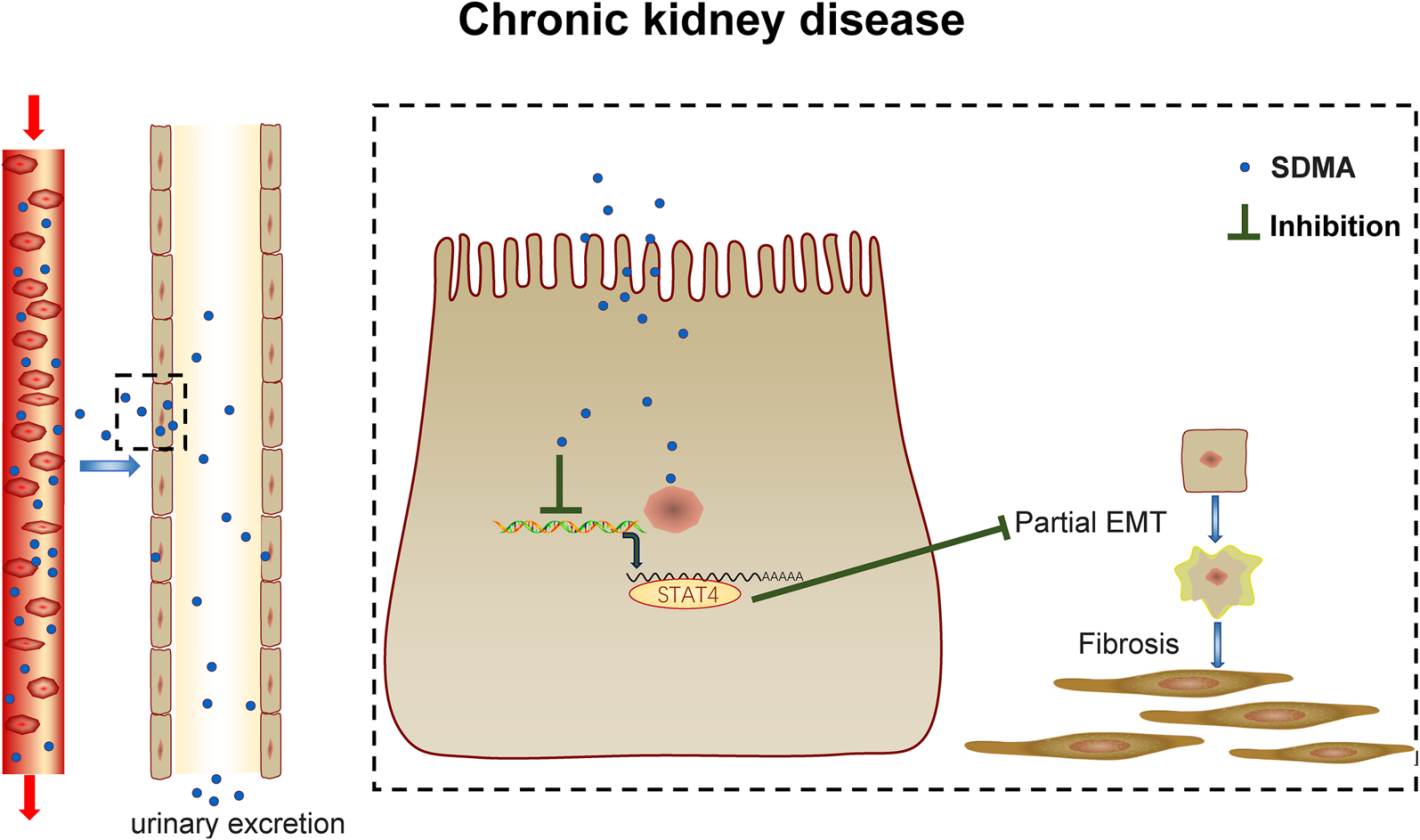
Schematic model of the role of SDMA in renal fibrosis. Circulating SDMA is excreted through renal tubule and enters renal epithelial cells to inhibit the expression of STAT4, and thus inhibits partial EMT of renal tubular cells and attenuates renal tubulointerstitial fibrosis in chronic kidney disease

SDMA inhibits partial EMT and attenuates renal tubulointerstitial fibrosis through inhibition of STAT4 (Fig. 6), suggesting that enhancing renal SDMA production or inhibition of STAT4 could be a new strategy to treat CKD patients with renal tubulointerstitial fibrosis.

## Supplementary Information

**Additional file 1: Figure S1.** Renal injection has no effect on normal kidneys. Male c57 mice received sham operation and were sacrificed at day 7. Renal injection of 50 μL of normal salineor 10 mM SDMAwas performed during sham operation. The left ureter was clamped for 30 min after renal injection. Renal fibrosis was assessed by Masson’s trichrome staining. Scale bar = 200 µm for upper figures and scale bar = 100 µm for lower figures. The expression of fibronectin, collagen-I, and α-SMA were analyzed by Western blottingand quantified. One representative of at least three independent experiments is shown. Data represent mean ± SD. NS represents not significant. ***p < 0.001.

**Additional file 2: Table S1.** List of primers used for quantitative PCR.

## Data Availability

The datasets used and/or analysed during the current study are available from the corresponding author on reasonable request.

## Abbreviations

SDMA: Symmetric dimethylarginine
UUO: Unilateral ureteral obstruction
UIRI: Unilateral ischemia–reperfusion injury
CKD: Chronic kidney diseases
ECM: Extracellular matrix
EMT: Epithelial to mesenchymal transition
ESRD: End stage renal disease
HK2: Human renal epithelial
PPARA: Peroxisome proliferative activated receptor alpha
HNF4A: Hepatocyte nuclear factor 4 alpha
NFκB, such as c-Rel: Nuclear factor kappa-B
SOX6: Sex determining region Y-Box 6
EGR2: Early growth response protein 2
KLFs: Krüppel-like factors
STAT4: Signal transducer and activator of transcription-4
pSmad3: Phosphorylated Smad3
NS: Normal saline
ADMA: Asymmetrical dimethylarginine
GO: Gene ontology
qPCR: Quantitative PCR
FBS: Fetal bovine serum
NC: Nonsense control
DEGs: Differentially expressed genes
ANOVA: One-way analysis of variance
