# Supplementary figures and images for: SDMA attenuates renal tubulointerstitial fibrosis through inhibition of STAT4
Source: J Transl Med. 2023 May 16;21:326. doi: 10.1186/s12967-023-04181-9 (PMC10186707; doi:10.1186/s12967-023-04181-9)

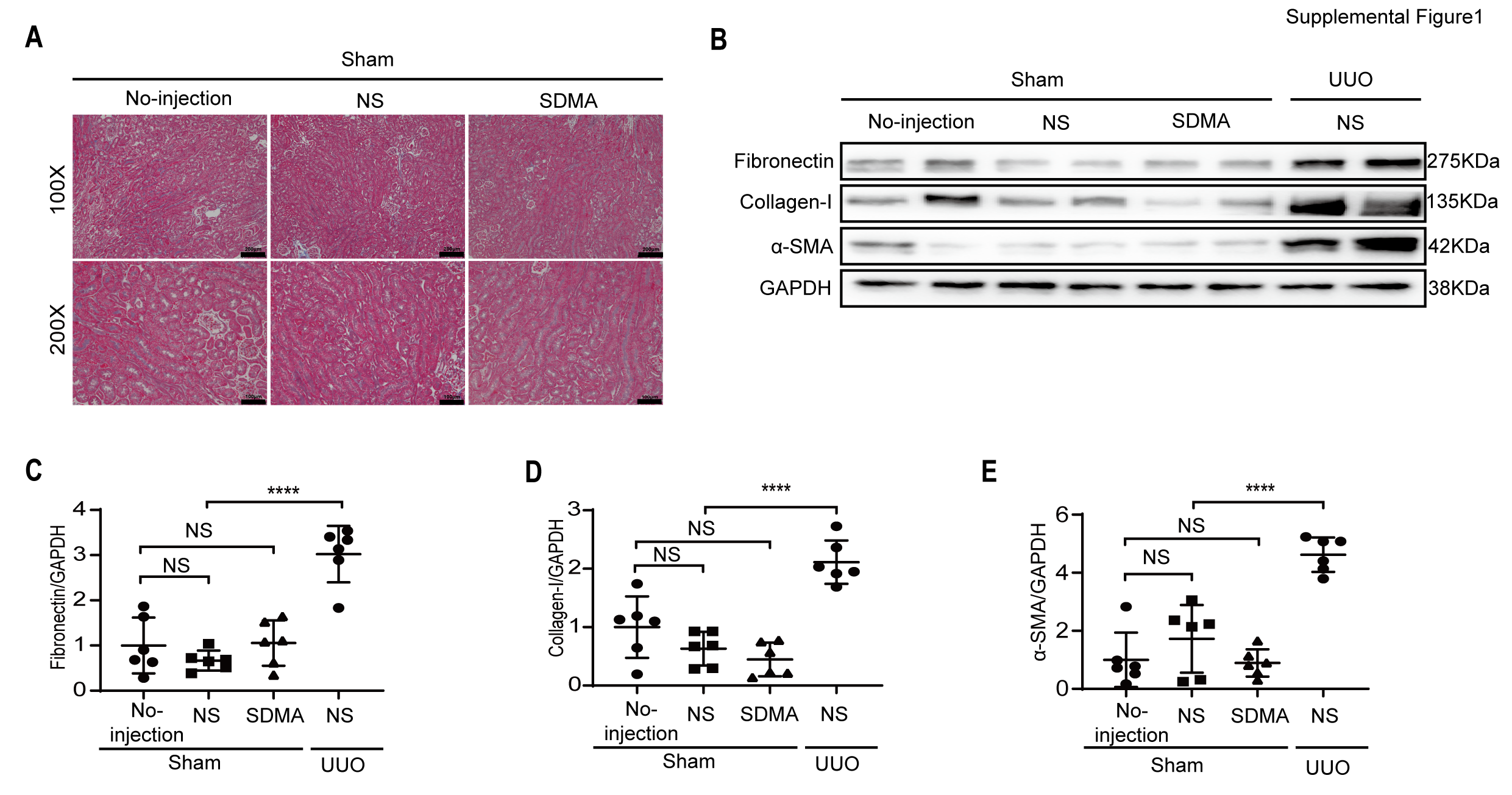

Supplement: Supplementary file 1 — Additional file 1: Figure S1. Renal injection has no effect on normal kidneys. Male c57 mice received sham operation and were sacrificed at day 7. Renal injection of 50 μL of normal salineor 10 mM SDMAwas performed during sham operation. The left ureter was clamped for 30 min after renal injection. Renal fibrosis was assessed by Masson’s trichrome staining. Scale bar = 200 µm for upper figures and scale bar = 100 µm for lower figures. The expression of fibronectin, collagen-I, and α-SMA were analyzed by Western blottingand quantified. One representative of at least three independent experiments is shown. Data represent mean ± SD. NS represents not significant. ***p < 0.001. [file 12967_2023_4181_MOESM1_ESM.tif]
