# Supplementary material for: SDMA attenuates renal tubulointerstitial fibrosis through inhibition of STAT4
Source: J Transl Med. 2023 May 16;21:326. doi: 10.1186/s12967-023-04181-9 (PMC10186707; doi:10.1186/s12967-023-04181-9)
Supplement: Supplementary file 2 — Additional file 2: Table S1. List of primers used for quantitative PCR. [file 12967_2023_4181_MOESM2_ESM.pdf]

Table S1: List of primers used for quantitative PCR

Primer Name	Forward	Reverse
**mouse Stat4**	CTCAGTAAGATGACGCAGATA	GCCAGTAGGGTAAAGCAGTT
**mouse Klf15**	CACCTGTCCCAGTCCAAAGC	AGGCTGGCTTCAGTTCTTCC
**mouse Pparα**	CGAAGACAAAGAGGCAGAGG	AGGAGGACAGCATCGTGAAG
**mouse Rel**	GAGGGGAATGCGGTTTAGATACAA	TGGGAGGCACAGCAGTTGTGAAGT
**mouse Sox6**	GCCCTTCCTCACCAGAAGAG	ATAAGGGCATGATCAAGGGG
**mouse Hnf4a**	CCCCCTCCTCCAATGGTACT	ACGCCTGATGCATTGAAGGT
**mouse Egr2**	ACTCTTCCAGCCTTCCTTCC	CGTACAGGTCTTTGCGGATG
**mouse Klf1**	CCTCCATCAGTACACTCACC	CCTCCGATTTCAGACTCACG
**mouse Klf6**	GAGAATTCAGCATGAAACTTTCACC TGCG	ACGCTCGAGCCTACAGGATTCGTC CCTC
**mouse Myb**	ATTGTGGACCAGACCAGACC	CTTCTTTCACGGAGTGGTCG
**mouse Gapdh**	AGGTCGGTGTGAACGGATTTG	TGTAGACCATGTAGTTGAGGTCA
